# Intratumoral diversity of telomere length in individual neuroblastoma tumors

**DOI:** 10.18632/oncotarget.2115

**Published:** 2014-06-18

**Authors:** Annalisa Pezzolo, Angela Pistorio, Claudio Gambini, Riccardo Haupt, Manuela Ferraro, Giovanni Erminio, Bruno De Bernardi, Alberto Garaventa, Vito Pistoia

**Affiliations:** ^1^ Laboratorio di Oncologia Istituto Giannina Gaslini Genova, Italy; ^2^ Epidemiologia e Biostatistica Istituto Giannina Gaslini Genova, Italy; ^3^ Laboratorio di Anatomia Patologica Istituto Giannina Gaslini Genova, Italy; ^4^ Dipartimento di Emato-Oncologia Pediatrica Istituto Giannina Gaslini Genova, Italy

**Keywords:** Telomere length, ALT mechanism, telomerase, neuroblastoma

## Abstract

The purpose of the work was to investigate telomere length (TL) and mechanisms involved in TL maintenance in individual neuroblastoma (NB) tumors. Primary NB tumors from 102 patients, ninety Italian and twelve Spanish, diagnosed from 2000 to 2008 were studied. TL was investigated by quantitative fluorescence *in situ* hybridization (IQ-FISH) that allows to analyze individual cells in paraffin-embedded tissues. Fluorescence intensity of chromosome 2 centromere was used as internal control to normalize TL values to ploidy. Human telomerase reverse transcriptase (hTERT) expression was detected by immunofluorescence in 99/102 NB specimens.

The main findings are the following: 1) two intratumoral subpopulations of cancer cells displaying telomeres of different length were identified in 32/102 tumors belonging to all stages. 2) hTERT expression was detected in 99/102 tumors, of which 31 displayed high expression and 68 low expression. Alternative lengthening of telomeres (ALT)-mechanism was present in 60/102 tumors, 20 of which showed high hTERT expression. Neither ALT-mechanism nor hTERT expression correlated with heterogeneous TL. 3) High hTERT expression and ALT positivity were associated with significantly reduced Overall Survival. 4) High hTERT expression predicted relapse irrespective of patient age. Intratumoral diversity in TL represents a novel feature in NB.

In conclusion, diversity of TL in individual NB tumors was strongly associated with disease progression and death, suggesting that these findings are of translational relevance. The combination of high hTERT expression and ALT positivity may represent a novel biomarker of poor prognosis that deserves further investigation.

## INTRODUCTION

Telomeres are specific DNA regions at the ends of chromosomes that prevent DNA damage and promote genomic stability [1, 2]. Telomeric DNA consists of tandem repeats of TTAGGG and is bound to a six subunit protein complex, referred to as shelterin or telosome, composed of TRF1, TRF2, TIN2, POT1, TPP1 and hRap1 [3]. Telomeres shorten with each round of DNA replication until a critical phase when they become dysfunctional, resulting in genomic instability [1-6]. Genomic alterations observed in cancers can be caused by inappropriate DNA repair at dysfunctional telomeres leading to chromosomal rearrangements, aneuploidy, and repression of DNA damage checkpoints [7]. To proliferate beyond the senescence checkpoint, cells must restore their telomere length (TL) [8]. Tumor cells maintain TL by reactivating human telomerase reverse transcriptase (hTERT), a ribonucleoprotein that catalyzes the synthesis and elongation of telomeres using an RNA template [8]. Moreover, an intratelomeric recombination mechanism known as alternative lengthening of telomeres (ALT) may be employed by tumor cells in order to ensure their replicative potential [9]. Telomeres in ALT cells are heterogeneous in length due to rapid deletions and elongations, which are thought to occur through high rates of inter-chromosomal recombination including a process termed telomere sister chromatid exchange (TSCE) [10]. In some primary tumors and cancer cell lines ALT-mechanism may substitute for or coexist with hTERT [11-13].

Neuroblastoma (NB), a pediatric malignancy of neuroectodermal origin, includes biologically and clinically heterogeneous tumors [14-15]. Tumor stage [14], age at diagnosis [18], tumor histology [19], *MYCN* amplification (MNA) [20], and ploidy [21] are the main prognostic factors for NB patients. MNA is strongly associated with rapid progression and poor prognosis irrespective of age and disease stage [22, 23].

hTERT is a prognostic marker in various adult and pediatric tumors, including NB [23-25], in which unchanged or long telomeres and high levels of telomerase expression/activity were found to predict poor outcome [26-29].

Here we have investigated TL and the involvement of hTERT and ALT-mechanism in TL maintenance in a series of NB tumors.

## RESULTS

### Diversity of Telomere Length in individual Neuroblastoma tumors

**Figure 1:**
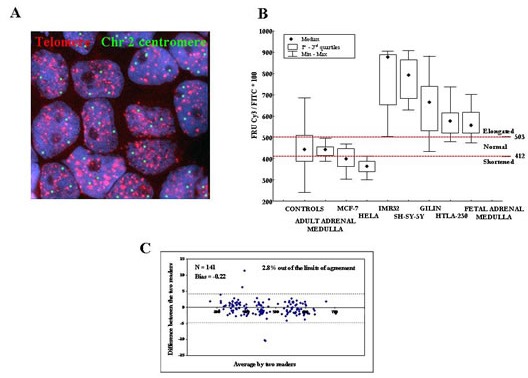
A: Interphase Quantitative Fluorescence Hybridization (IQ-FISH) using pan-telomere (red) and chromosome 2 centromeric (green) peptide nucleic acid (PNA) probes in paraffin embedded NB tissue section The nuclei are stained with DAPI (blue). Magnification x100. B: Calibration of IQ-FISH for TL measurements using four NB cell lines (IMR32, SHSY-5Y, GILIN and HTLA-230) and fetal adrenal medulla as long telomere controls, HeLa and MCF-7 cell lines as short telomere controls, peripheral blood mononuclear cells from adult healthy donors as well as adult adrenal medulla as normal telomere controls. We defined the minimum and maximum cut-off values of fluorescence ratio units (FRU) as 412 and 503, respectively. C: FRU values analyzed by two readers by means of the Bland and Altman's plot. Except for four data points (4/141; 2.8%) all values fell within 95% of the limits of agreement.

The IQ-FISH procedure on tissue sections is a suitable approach for the assessment of TL in relation to cell type, and in the context of tissue architecture [30-36]. TL was measured in 102 primary NB tumors by a modified IQ-FISH assay with ploidy correction that showed high sensitivity (0.1 kb of telomere repeats) and accuracy (99%) (Fig 1 A-C). Seventy NB cases (68.6%) displayed homogeneous TL, of which 42 were short, 25 long, and 3 normal. In the remaining 32 NB cases (31.4%), single cell analysis revealed the coexistence in the same tumor of two cancer cell subpopulations with differing TL, and namely i) normal and short telomeres, or ii) long and short telomeres, or iii) normal and long telomeres. These 32 NB cases displaying heterogeneous TL showed stage and age distribution, risk group, favorable or unfavorable histology, frequency of MNA and ploidy comparable to cases with homogeneous TL (Table S1).

**Figure 2:**
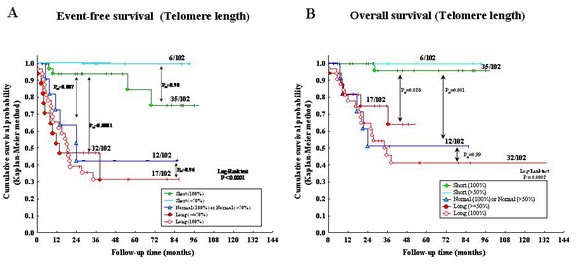
A: Kaplan-Meier Event-Free Survival curve for five patient groups based upon telomere length (TL) B: Kaplan-Meier Overall Survival curve for five groups based upon TL.

The 102 NB samples were arbitrarily classified in five groups based upon TL: 1) 100% short TL (n=35); 2) higher than 50% short TL (n=6); 3) 100% normal TL or higher than 50% normal TL (n=12); 4) higher than or equal to 50% long TL (n=17); 5) 100% long TL (n=32). NB cases belonging to group 5 had significantly lower EFS (Figure 2A) and OS (Fig 2B) than cases belonging to group 1 after Bonferroni's correction (P_B_<0.0001 and P_B_<0.0001, respectively).

NB cases with heterogeneous TL belonging to group 2 (i.e. predominance of short TL) had EFS and OS super-imposable to those of cases with homogeneously short TL belonging to group 1 (P_B_=0.90 and 0.99, respectively) (Fig 2A and 2B). Likewise, NB cases with heterogeneous TL belonging to group 4 (i.e. predominance of long TL) had EFS and OS super-imposable to those of cases with homogeneously long TL belonging to group 5 (P_B_=0.96 and 0.99, respectively) (Fig 2A and 2B).

In contrast, significantly better EFS and OS were detected in cases with short (group 1) *vs* normal (P_B_=0.007 and 0.001, respectively) (group 3) or *vs* long (group 4) TL (P_B_<0.0001 and 0.028, respectively). Finally, EFS and OS curves of cases with heterogeneously (group 4) or homogeneously (group 5) long TL were super-imposable (P_B_=0.96 and 0.99, respectively) (Fig 2A and 2B).

Based upon the above results, cases belonging to groups 1 and 2 (i.e. short TL) or to groups 3, 4 and 5 (i.e. long/normal TL) were clustered in two groups in order to assess the prognostic impact of hTERT expression and ALT mechanism in relation to TL (see below).

### Detection of ALT and h-TERT in tumor tissues

**Figure 3:**
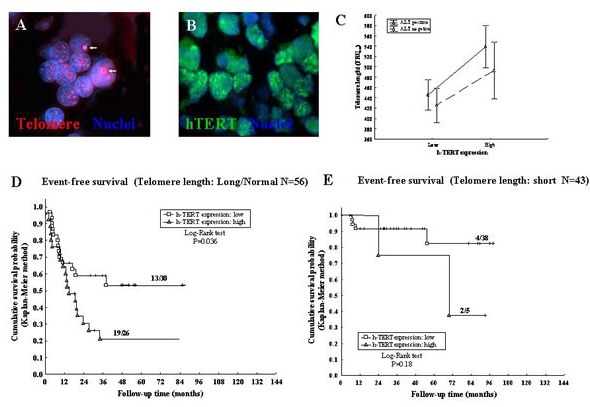
A: ALT positive NB cells showing ALT-associated bright intra-nuclear foci of telomere FISH signals (red) (arrows) Nuclear DNA was counterstained with DAPI (blue). B: Immunofluorescence nuclear labeling for the catalytic subunit of telomerase hTERT (green). C: Variance analysis of TL in relation to presence or absence of ALT and to high or low hTERT expression. D: Kaplan-Meier Event-Free Survival curve for long/normal TL and hTERT expression. E: Kaplan-Meier Event-Free Survival curve for short TL and hTERT expression.

Telomere elongation is operated by telomerase and ALT mechanism, that is based on recombination of telomeric sequences and might cause heterogeneous TL in single cancer cells [10-12]. ALT was detected by FISH analysis [37-39] in 60/102 NB samples tested; one representative experiment is shown in Fig 3A. Sixteen out of 32 (50%) tumors with heterogeneous TL and 44/70 (62.9%) tumors with homogeneous TL were ALT positive (P=0.22). Variable expression of hTERT was detected by immunofluorescence in 99/102 (97%) NB specimens tested (Fig 3B). Thirty-one out of 99 (31.3%) tumors displayed high hTERT expression [(>0.398 units of fluorescence intensity (FI) while 68/99 (68.7%) showed low hTERT expression (≤0.398 FI). Twenty of 31 cases with high hTERT expression (64.5%) and 40/68 with low hTERT expression (58.8%) were ALT positive, indicating lack of correlation between ALT mechanism and hTERT expression levels (P=0.59).

The weighted means of the FRU values detected in each of the 32 tumors showing heterogeneous TL were used for the analyses reported in Fig 3 C, D, and E, as well as for Cox regression model. Two-ways analysis of TL variance in relation to presence or absence of ALT and high or low hTERT expression showed that the two mechanisms of telomere elongation operated independently each other (Fig 3C). A statistically non significant trend to longer telomeres in ALT positive *vs* ALT negative cases independent of the levels of hTERT expression was observed (Fig 3C).

### Event-Free and Overall Survival analysis

EFS and OS analyses, with details about incidence rates of relapses and/or death, with 95% CI, HRs and P values are shown in Table 1. There was a significant relationship between all variables reported in Table 1 and the occurrence of relapse or death. In particular, normal TL patients showed higher incidence rate of relapse/death (27.7× 1000 *pm*) with respect to short TL patients (3.3 1000 *pm*) (P<0.0001); the same higher incidence rate was observed in long TL patients (22.7× 1000 *pm*).

The same variables that were related to EFS influenced OS, with the exception of ploidy that was not statistically significant. In contrast, ALT-mechanism emerged as a significant prognostic factor since OS was reduced in ALT positive *vs* ALT negative patients (P=0.035) (Table 1).

### Prognostic impact of hTERT expression and ALT mechanism in relation to Telomere Length

Positivity or negativity for ALT-mechanism had no effect on TL-related EFS, as assessed by Kaplan-Meyer analyses (Table 1). EFS of NB patients with long/normal TL (Fig 3D) was significantly reduced when hTERT expression was high *vs* low (P=0.036), whereas EFS of patients with short TL (Fig 3E) was unaffected by hTERT expression levels (P=0.18). We next subdivided NB patients into four groups based upon positivity/negativity for ALT and high/low hTERT tumor expression, i.e. i) hTERT low/ALT-negative, ii) hTERT low/ALT-positive, iii) hTERT high/ALT-negative, and hTERT high/ALT-positive.

**Table 1:** Incidence Rates and Hazard Ratios (HR) for Event-Free Survival (EFS) and Overall Survival (OS); (N=102)

	N	Number (%) of relapses or deaths	Incidence Rates x 1000 (EFS)	HR (P)	Number (%) of deaths	Incidence Rates × 1000 (OS)	HR (P)
Age							
≥ 18 months	44	25 (56.8%)	19.4 (13.1-28.7)	2.45 (P=0.017)	19 (43.2%)	12.3 (7.9-19.3)	3.2 (P=0.006)
< 18 months (reference)	58	16 (27.6%)	7.9 (4.8-12.9)		9 (15.5%)	3.9 (2.0-7.5)	
Stage							
4	31	20 (64.5%)	19.2 (12.4-29.8)	2.1 (P=0.004)	18 (58.1%)	15.1 (9.5-24.0)	4.0 (P<0.0001)
1, 2, 3, 4s (reference)	71	21 (29.6%)	9.2 (6.0-14.2)		10 (14.1%)	3.7 (2.0-7.0)	
MYCN status							
Amplified	44	25 (56.8%)	22.6 (15.3-33.4)	3.1 (P=0.004)	21 (47.7%)	17.1 (11.2-26.2)	6.4 (P<0.0001)
Not amplified (reference)	58	16 (27.6%)	7.25 (4.4-11.8)		7 (12.1%)	2.7 (1.3-5.6)	
Ploidy							
Diploid	49	26 (53.1%)	16.6 (11.3-24.4)	1.9 (P=0.029)	17 (34.7%)	9.0 (5.6-14.5)	1.6 (P=0.10)
Hyperdiploid (reference)	53	15 (28.3%)	8.6 (5.2-14.2)		11 (20.7%)	5.6 (3.1-10.1)	
Histology							
Unfavorable histology	50	30 (60.0%)	19.3 (13.5-27.7)	3.1 (P=0.001)	24 (48.0%)	13.2 (8.8-19.6)	6.7 (P<0.0001)
Favorable histology (reference)	52	11 (21.1%)	6.2 (3.45-11.3)		4 (7.7%)	2.0 (0.7-5.2)	
Risk Group (N=86)							
High/Intermediate	58	33 (56.9%)	19.4 (13.5-27.7)	4.8 (P=0.002)	27 (46.6%)	13.8 (9.5-20.1)	15.2 (P=0.0003)
Low (reference)	28	4 (14.3%)	4.0 (1.5-10.7)		1 (3.6%)	0.9 (0.1-6.5)	
Telomere length							
Long	46	27 (58.7%)	22.7 (15.6-33.2)	7.0 (P<0.0001)	19 (41.3%)	12.2 (7.8-19.1)	7.8 (P=0.0006)
Normal	13	8 (61.5%)	27.75 (13.9-55.5)	8.5	6 (46.1%)	16.0 (7.2-35.6)	10.2
Short (reference)	43	6 (13.9%)	3.26 (1.5-7.3)		3 (7.0%)	1.6 (0.5-4.8)	
h-TERT (N=99)							
> 0.398 (> 0.205)	31	21 (67.7%)	26.1 (17.0-40.1)	3.8 (P<0.0001)	23/61 (37.7%)	10.6 (7.0-16.0)	5.9 (P=0.003)
≤ 0.398 (≤ 0.205)	68	17 (25.0%)	6.8 (4.3-11.0)		3/38 (7.9%)	1.8 (0.6-5.7)	
ALT mechanism							
Positive	60	28 (46.7%)	15.1 (10.4-21.9)	1.7 (P=0.11)	21 (35.0%)	9.5 (6.2-14.5)	2.2 (P=0.035)
Negative	42	13 (30.9%)	8.9 (5.2-15.3)		7 (16.7%)	4.3 (2.0-9.0)	

P values refer to the Log-Rank test.

**Figure 4:**
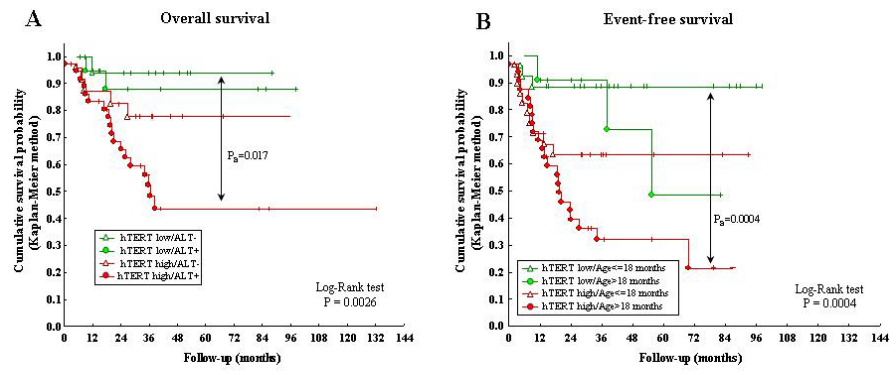
A: Kaplan-Meier Overall Survival curve for ALT positivity/negativity and hTERT high/low expression B: Kaplan-Meier Event-Free Survival curve for hTERT high/low expression and age at diagnosis (> or ≤18 months).

hTERT expression combined with ALT-mechanism influenced significantly OS, as assessed by Kaplan-Meier analyses (P=0.0026) (Fig 4A). Thus, NB patients with high hTERT/ALT positivity showed significantly lower OS than patients with low hTERT/ALT-negativity (P_B_=0.017). Finally, OS of patients with low hTERT expression did not significantly differ in ALT positive *vs* negative cases.

Since age is the most important prognostic factor in NB [18], we next evaluated EFS of the following patient groups, i) hTERT low/age ≤18 months, ii) hTERT low/age >18 months, iii) hTERT high/age ≤18 months, and iv) hTERT high/age >18 months. Patients older than 18 months with high hTERT had a significantly reduced EFS compared to patients <18 months with low hTERT (P_B_ =0.0004) (Fig 4B).

Finally, we performed best fitted Cox regression model for EFS and OS of our NB patient cohort. Long or normal TL was the best independent predictor of relapse (P=0.003), followed by tumor stage 4 (P=0.03) and high hTERT expression (P=0.03). Stage 4 and MNA were the best independent predictors of relapse (P<0.0001 and 0.0002, respectively), followed by high hTERT expression (P=0.002) (Table 2).

**Table 2:** Best fitted Cox regression model for Event-Free Survival (EFS) and Overall Survival (OS)

	HRAdj	(95% CI)	P
Event-Free Survival (EFS) (n. events/total: 38/99; 38.4%)			
Telomere length: Long vs Short (reference)	4.0	1.6 – 10.3	0.003
Normal vs Short (reference)	4.3	1.4 – 13.4	
Stage = 4 vs 1, 2, 3, 4s (reference)	2.1	1.1 – 4.0	0.03
h-TERT: > 0.398 vs ≤ 0.398 (reference)	2.1	1.1 – 4.1	0.03
			
Overall Survival (OS) (n. events/total: 26/99; 26.3%)			
Stage = 4 vs 1, 2, 3, 4s (reference)	5.4	2.3 – 12.5	<0.0001
MYCN status: Amplified vs Not amplified (reference)	4.6	1.9 – 11.1	0.0002
h-TERT: > 0.205 vs ≤ 0.205 (reference)	5.7	1.7 – 19.1	0.002

P values refer to the Log-Likelihood Ratio test.

## DISCUSSION

We have shown that one third of NB tumors contained two cancer cell subpopulations with different TL, as assessed by NB-tailored IQ-FISH with data normalization for DNA ploidy [33]. This latter step is critical to minimize misinterpretations due to the abnormal number of chromosomes possibly present in the nucleus of cancer cells. Our results are consistent with a model whereby genomic crisis generated due to telomere attrition induces subclonal heterogeneity, potentially leading to TL heterogeneity [40]. The translational impact of the results obtained is highlighted by the finding that patients with predominantly (>50%) long/normal TL had the same unfavorable EFS and OS as patients with homogenously long/normal TL. Thus, the former patients might belong to a novel risk category. Failure of previous studies on NB patients to identify heterogeneous TL in individual tumors likely depends on the need for i) single cells analysis using methods as IQ-FISH or flow FISH, and ii) ploidy normalization.

Telomeres in ALT cells are highly heterogeneous in length and are maintained through a mechanism involving recombination [10], whose pathway remains to be elucidated. We investigated ALT-mechanism and hTERT expression, that correlates with the catalytic activity of telomerase [24], in NB cases with heterogeneous *vs* homogeneous TL. ALT-mechanism, that was detected in more than half of the tumors investigated, was unrelated to TL. Other investigators have correlated ALT-mechanism with long TL in NB, but the patient groups analyzed were too small to draw any definitive conclusion [29].

In our study, ALT-mechanism and hTERT operated independently each other. Presence or absence of ALT-mechanism had no prognostic relevance in NB cases with low hTERT expression, but coexistence of high hTERT and ALT reduced significantly OS compared to cases with high hTERT and absence of ALT. These latter findings suggest that hTERT and ALT may cooperate in promoting NB progression.

Here we show for the first time that ALT-mechanism and hTERT were co*-*expressed in approximately 60% of individual NB tumors. Whether ALT-mechanism and hTERT expression occur in mutually exclusive tumor cell subsets or rather in the same cell population warrants further investigation. In this respect, ALT-mechanism and hTERT expression were detected in discrete subpopulations of primary osteosarcoma cells [41].

Cox regression analysis showed that high hTERT expression was a robust independent predictor of EFS and OS for our NB patients, consistent with most, but not all, previous reports [27, 41-44]. Nonetheless, we found that high hTERT expression showed only a moderate correlation with TL (R=0.48), suggesting that the unfavorable prognosis of NB patients with high hTERT expression may be related to telomerase functions other than telomere elongation [6], such as i) transcriptional modulation of Wnt/β-catenin signaling pathway [45]; ii) enhancement of cell proliferation and/or resistance to apoptosis [45]; iii) involvement in DNA-damage repair [46]; iv) activity as RNA-dependent RNA polymerase [47]. Moreover, when telomeres become critically short, they activate a DNA damage response and trigger the induction of replicative cellular senescence that can be suppressed by over-expression of hTERT [3].

We finally investigated the prognostic impact of high/low hTERT expression in relation to age at diagnosis lower or higher than 18 months [18]. Patients older than 18 months with high hTERT expression had worse EFS than patients of the same age with low hTERT expression. Likewise, patients younger than 18 months with high hTERT expression showed worse EFS than patients of the same age group with low hTERT expression. Taken together, these results demonstrate that high hTERT expression represents an unfavorable prognostic factor irrespective of patient age.

In conclusion, diversity of TL in individual NB tumors was strongly associated with disease progression and death. High hTERT associated with ALT-mechanism may represent a novel biomarker of poor prognosis.

## METHODS

### Patients and Clinical Follow-up

A retrospective series of primary tumors from 102 NB patients was collected at the Istituto Giannina Gaslini, Genova (90 patients), Italy and at the Medical School of the University of Valencia, Spain (12 patients), from January 2000 to December 2008. Table S1 shows the demographic characteristics of the patients investigated. The study was approved by the Institutional Review Boards of the two participating Institutions and informed consent was obtained from patients or their legal guardians.

Patients were classified according to the International Neuroblastoma Staging System [14] and to the International NB Risk Group (INRG) [22] classifications. Eligibility criteria for inclusion in the analytic cohort were diagnosis of *bona fide* NB and lack of any treatment at study. Twenty-eight patients died of disease. Seventy-four survivors were followed-up and categorized at the time of their last clinical examination. Clinical follow-up was performed for all patients with a median follow-up time of 3.6 years, with a minimum follow-up duration, in surviving patients, of 3.1 months. Event-Free Survival (EFS) was calculated from diagnosis to last follow-up or event (first occurrence of relapse, progression, or death). Overall Survival (OS) was calculated from diagnosis to last follow-up or death.

### Tumor Specimens

Formalin-fixed, paraffin-embedded tissue sections from 102 NB tumors were studied. Each tumor area tested for TL contained malignant cells, as assessed by histological examination. Quantification of telomere fluorescence intensity was performed on serial tumor tissue sections, thus allowing telomere quantification in tumor areas selected by the pathologist. Tumor cells were distinguished in the samples using NB-specific marker NB84 [47]. All tumors were evaluated at the time of diagnosis prior to any treatment other than surgery.

### Telomere Length Measurement

Interphase Quantitative Fluorescence *In Situ* Hybridization (IQ-FISH) [30-36] was performed on 4-μm-thick paraffin-embedded NB tissue sections. The Cy3-labeled PNA probe specific for telomeric sequences (Telomere PNA FISH Kit/Cy3, Dako) and, as internal reference, the FITC-labelled PNA probe specific for chromosome 2 centromere (kindly provided by Dako), were used. To define cut-off values the following samples were tested, i) peripheral blood mononuclear cells (PBMC) from nine human healthy subjects, obtained after informed consent and isolated on Ficoll-Hypaque gradients, ii) the MCF7 and HeLa human tumor cell lines with known TL of 4.07 Kb 3.44 Kb, respectively [32], iii) cytospins of PBMC fractions and IMR32, SHSY-5Y, GILIN and HTLA-230 NB cell lines that had been fixed in 0.5% paraformaldehyde for 20 min to simulate formalin-fixed tissues [31, 32], and iv) paraffin-embedded tissue sections from adult and fetal adrenal medulla.

Telomere and centromere fluorescence signals were automatically quantified on serial tumor tissue sections selected by the pathologist by using the fluorescence-based microscopic scanning system E-1000 Nikon with appropriate filters set and a high-resolution CCD camera, and the image analysis software Genikon (Nikon, Tokyo, Japan). A nuclear area for each cell was manually selected by the operator to measure centromere and telomere fluorescence intensities in the FITC and Cy3 images, respectively (Fig 1A). These latter fluorescence intensities were analyzed by scanning three consecutive serial images in order to avoid the loss of portions of the nucleus. The slide scanning and cell analysis procedures were performed by using a 100x objective (Nikon). We measured Cy3 pan-telomeric probe and chromosome 2 FITC centromeric probe fluorescence signal intensities in single nuclei and expressed the ratio between the former and the latter intensity values arbitrary fluorescence ratio units (FRU). A minimum of 20 nuclei were scanned and the mean value of the FRU was calculated. FRU values corresponded to TL and were corrected for ploidy as reported [33].

In order to define cut-off points for TL measurement by IQ-FISH, NB cell lines (IMR32, SHSY-5Y, GILIN and HTLA-230) [27] and fetal adrenal medulla samples were used as long telomere controls (coded as 1), HeLa and MCF-7 cell lines as short telomere controls [34], PBMCs from adult healthy donors and adult adrenal medulla as normal telomere controls. We determined the minimum and maximum cut-off values of FRU as 411.9 and 503.3, respectively (Fig 1B). Cut-off points were determined by means of the ROC curve analysis in two steps; the first cut-off was determined defining as “abnormally long” TL populations coded as 1, and the remaining cells lines (short and normal) coded as 0, obtaining, in the first ROC curve, the value of 503.3. The second cut-off was determined defining as “abnormally short” HeLa and MCF-7 cell lines (coded as 1) *versus* all the remaining cell lines, obtaining the value of 411.9.

### Reproducibility of Telomere Length Measurement

#### Effects of the fixation procedure on determination of FRU

To evaluate the potential deleterious effect of nuclear truncation induced by cut sections [31, 32] we compared TL assessed by IQ-FISH on intact nuclei from four paraformaldehyde-fixed tumor touch preparations and four paired paraffin-embedded tissue sections to simulate standard pathology slide preparation procedures. IQ-FISH gave strong nuclear signals on both tissue sections and touch preparations. The IQ-FISH coefficient of variation (CV) ranges were 5.79%-8.95% for formalin-fixed tissue sections and 3.3%-13.9% for paraformaldehyde-fixed touch preparations. These differences were not significant, indicating that the fixation procedure did not affect FRU.

#### Inter-assay variation

To estimate the reproducibility of IQ-FISH, serial tissue sections from the same specimen were processed in different experiments at different time points. The best CV observed was 2.3% and the worst was 15.5% with a median CV of 7.1%, indicating a good reproducibility of the assay.

#### Telomere Length Measurement by two independent investigators

We compared the FRU values (i.e. the ratio between telomere and centromere fluorescence intensities indicating TL) of all specimens analyzed and of all controls determined by two investigators using the Bland and Altman's plot. Except for four data points (4/141; 2.8%), all values fell within 95% of the limits of agreement (Fig 1C) and the bias value was good (Bias=−0.22). The Intra-class Correlation Coefficient was excellent (ICC=0.999).

### ALT assessment

ALT mechanism was detected as large ultra-bright intra-nuclear foci of telomere FISH signals [37-39] following hybridization with Cy3 pan-telomeric probe. Tumors containing ≥1% tumor cells displaying ALT-associated telomeric foci were considered ALT-positive [37-39].

### Quantification of hTERT Expression

Paraffin-embedded NB tissue sections were stained overnight at 4° C using indirect immunofluorescence with a monoclonal antibody anti-hTERT (1:100; Lab Vision, Fremont, CA, USA). The slides were incubated with FITC-labeled secondary antibody (1:1000) at 37° for 1 h. hTERT fluorescence intensity was quantified using the Genikon software. Results were expressed as mean fluorescence intensity (FI) from at least 20 nuclei.

### Statistical Analysis

Descriptive statistics were firstly performed and data were reported in terms of median values and 1^st^ and 3^rd^ quartiles (1^st^ – 3^rd^ q) for quantitative variables, in terms of absolute frequencies and percentages for categorical variables. The IQ-FISH inter-assay variation was estimated calculating the coefficient of variation (CV), i.e. standard deviation divided by the mean Fluorescence Ratio Unit (FRU) and multiplied by 100. The FDA recommended limit for CV% is <15% [48]. Bland and Altman's plot was used to assess the agreement between the two investigators' readings of FRU; this plot shows the differences of the two measurements (Y-axis) with respect to the their means (X-axis). The bias (mean of all differences) should be close to zero. Moreover the agreement between the two readings was evaluated by means of the Intra-class Correlation Coefficient (ICC) [49].

Categorical data were reported in terms of absolute frequencies and percentages (Table 1) and compared by the Chi-square test or by the Fisher's Exact test whenever expected frequencies were less than 5.

Receiver operating characteristic (ROC) curves were used to determine the best cut-off point for defining high/low h-TERT expression using event-free status as the main outcome variable; a value=0.398 was obtained; a second cut-off point for h-TERT expression (obtained by the same ROC curve method) was calculated for OS (considering only life status as outcome of interest); in this case a value=0.205 was obtained. Two-ways analysis of variance was used to evaluate telomere length in relation to presence/absence of ALT and high/low hTERT expression. As some tumors showed heterogeneous TL, the weighted mean of the FRU values was calculated and used for the analyses reported in Fig 3 C, D, and E, as well as in survival analyses.

EFS and OS curves were drawn categorizing for a series of demographic and clinical variables; these curves were estimated using the Kaplan-Meier method and compared by the log-rank test with P<0.05 considered statistically significant.

For each category of the demographic and clinical variables, the absolute number of relapses or deaths, the incidence rates expressed × 1000 person-months (pm) with 95% Confidence Intervals (95% CI), Hazard Ratios (HRs) and statistical significance obtained from the Log Rank test were calculated and reported. Factors significantly associated with higher probability of observing relapse or death were then tested in a Cox proportional hazards regression model. The Log-Likelihood Ratio test (LR test) was used for comparisons.

The statistical packages used were the Statistica (version 9.0, StatSoft Corp., Tulsa, OK, USA) for bivariate analyses and the Stata release 7 (Stata Corporation, Texas, USA) for multivariate analyses.

## SUPPLEMENTARY MATERIAL, TABLES


