# Supplementary material for: Intratumoral diversity of telomere length in individual neuroblastoma tumors
Source: Oncotarget. 2014 Jun 18;6(10):7493–503. doi: 10.18632/oncotarget.2115 (PMC4480695; doi:10.18632/oncotarget.2115)
Supplement: Supplementary file 1 [file oncotarget-06-7493-s001.pdf]

Intratumoral diversity of telomere length in individual Neuroblastoma tumors

Supplementary Material

Demographic and clinical characteristics of NB patients (N=102) and type of TL (heterogeneous/homogeneous)

	**TL: “heterogeneous”**	**TL: “homogeneous”**	**P**
**Age:**			
≥ 18 months	13/44 (29.6%)	31/44 (70.4%)	0.73
< 18 months	19/58 (32.8%)	39/58 (67.2%)	
**Stage:** 1	6/13 (46.2%)	7/13 (53.8%)	0.054§
2A/2B	13/18 (72.2%)	5/18 (27.8%)	
3	16/28 (57.1%)	12/28 (42.9%)	
4	27/31 (87.1%)	4/31 (12.9%)	
4s	7/12 (58.3%)	5/12 (41.7%)	
***MYCN* status:**			
Amplified	15/44 (34.1%)	29/44 (65.9%)	0.61
Not amplified	17/58 (29.3%)	41/58 (70.7%)	
**Ploidy:**			
Diploid	16/49 (32.6%)	33/49 (67.4%)	0.79
Hyperdiploid	16/53 (30.2%)	37/53 (69.8%)	
**Histology:**			
Unfavorable histology	16/50 (32.0%)	34/50 (68.0%)	0.89
Favorable histology	16/52 (30.8%)	36/52 (69.2%)	
**Risk Group (N=86):**			
High/Intermediate	18/58 (31.0%)	40/58 (69.0%)	0.92
Low	9/28 (32.1%)	19/28 (67.9%)	

P values refer to the Pearson’s Chi-square test unless otherwise specified; § Fisher’s Exact test.
